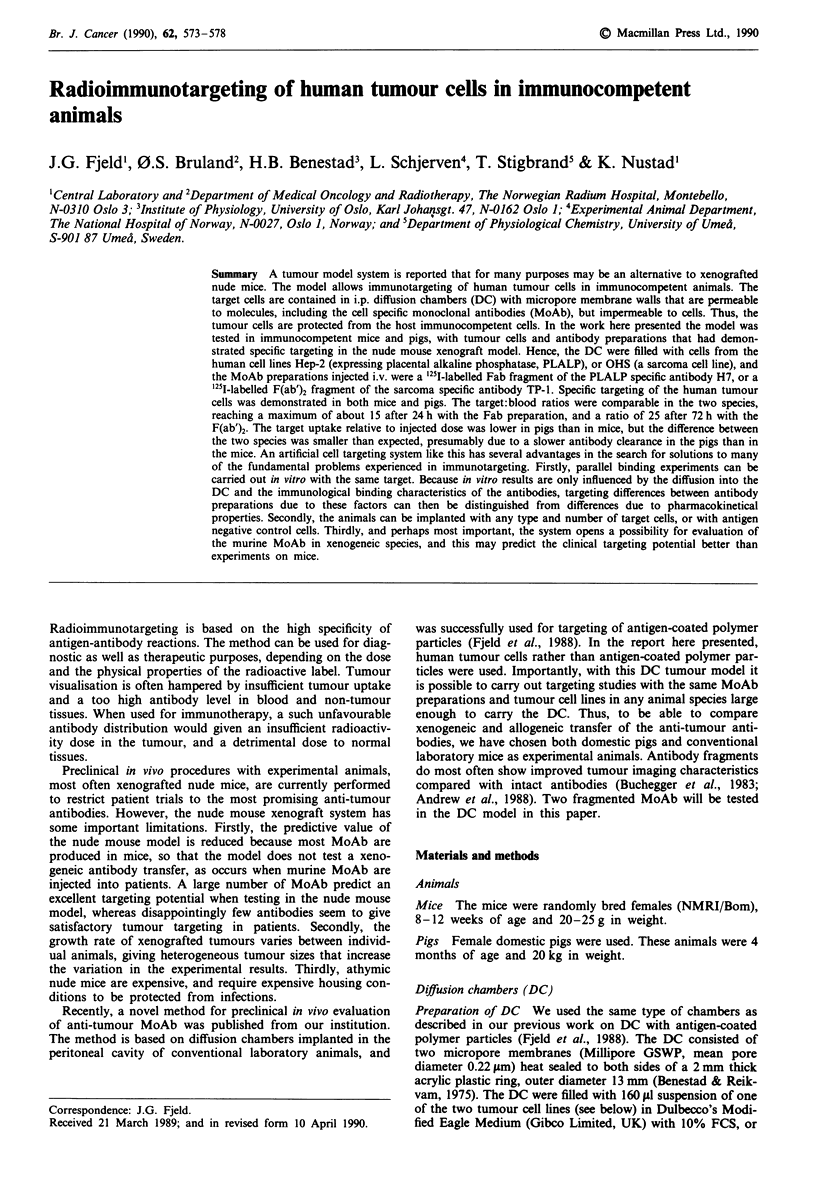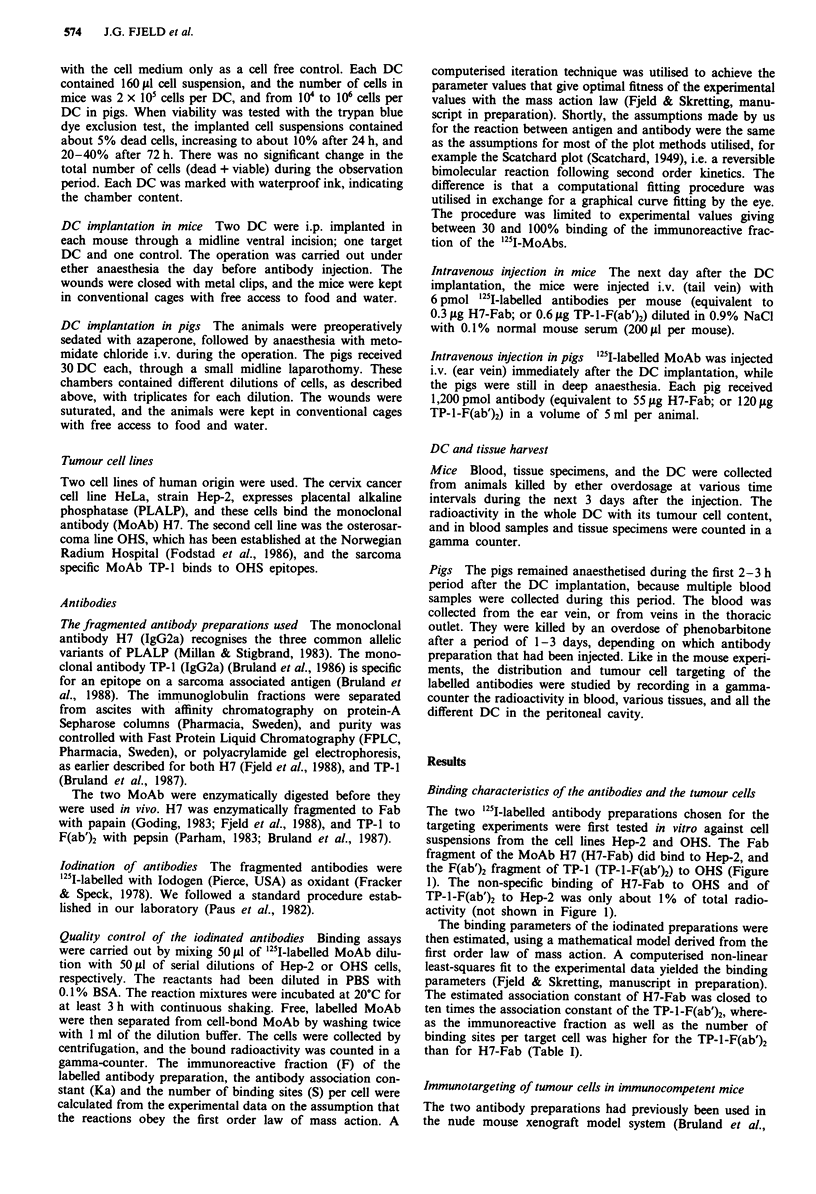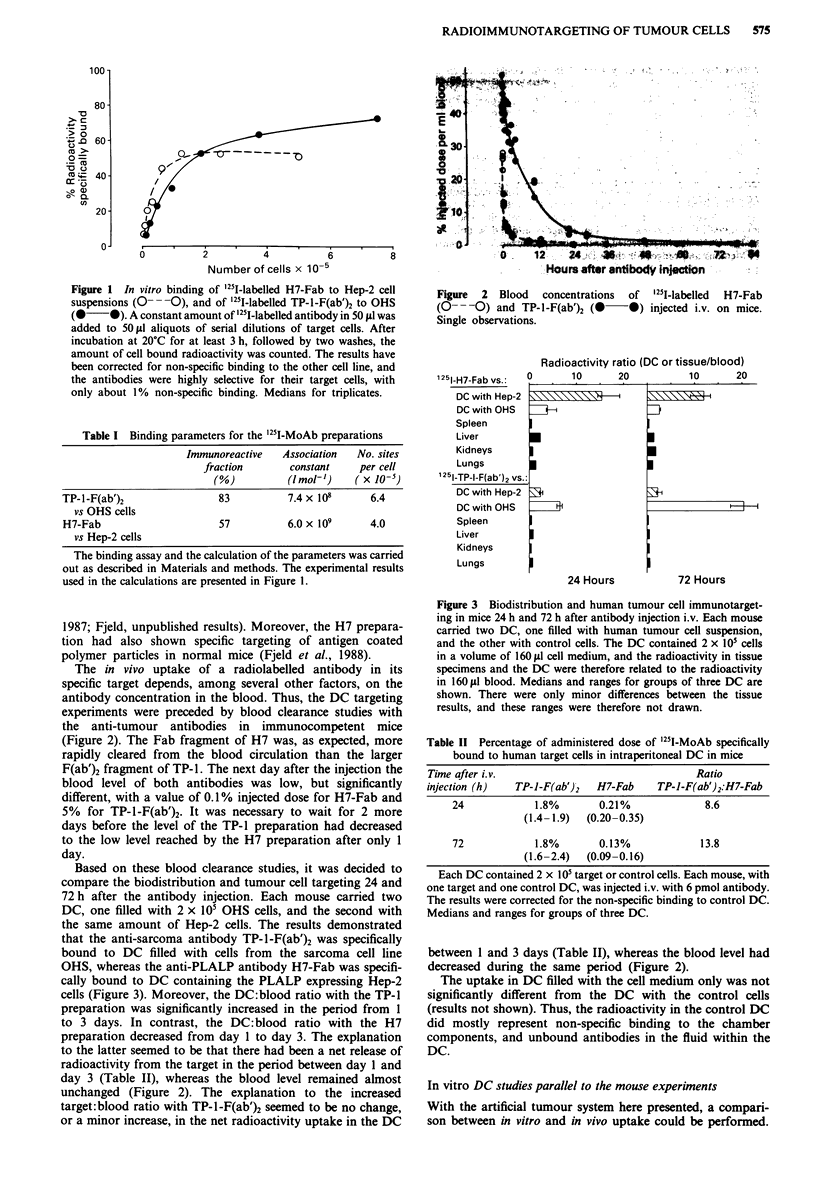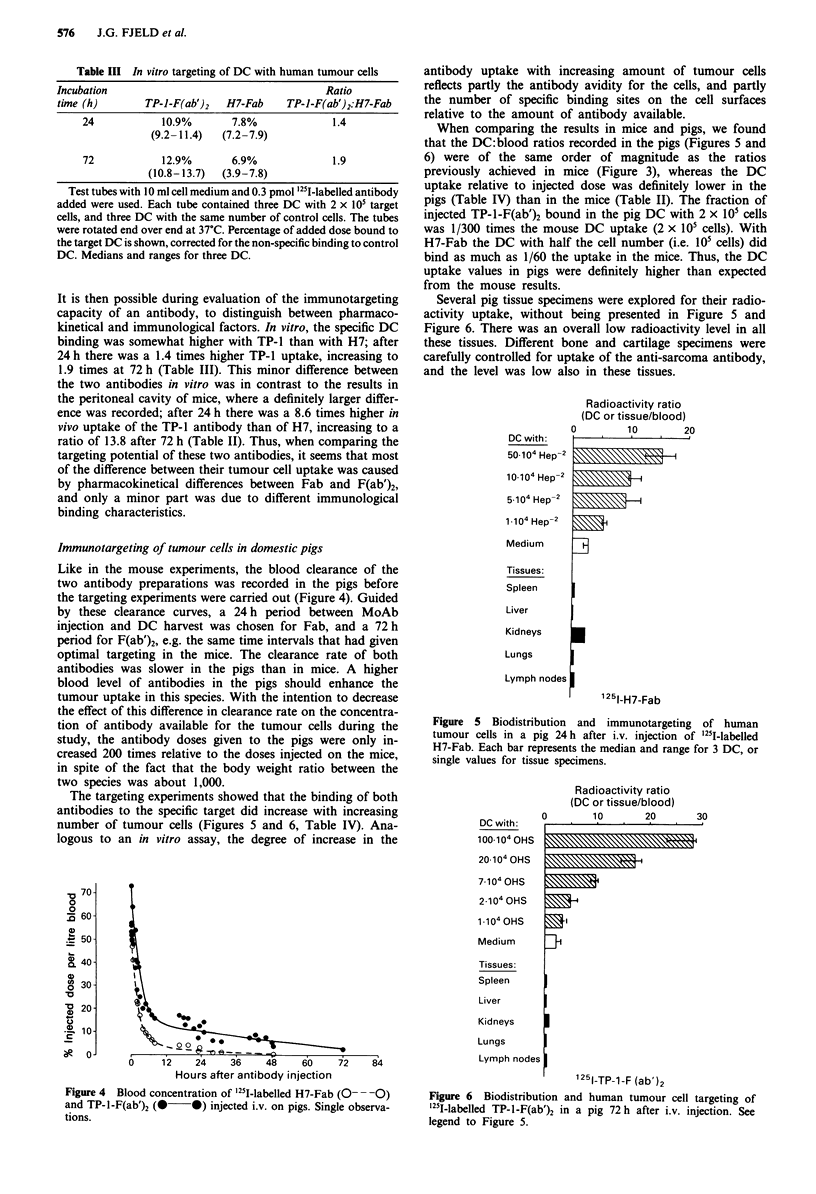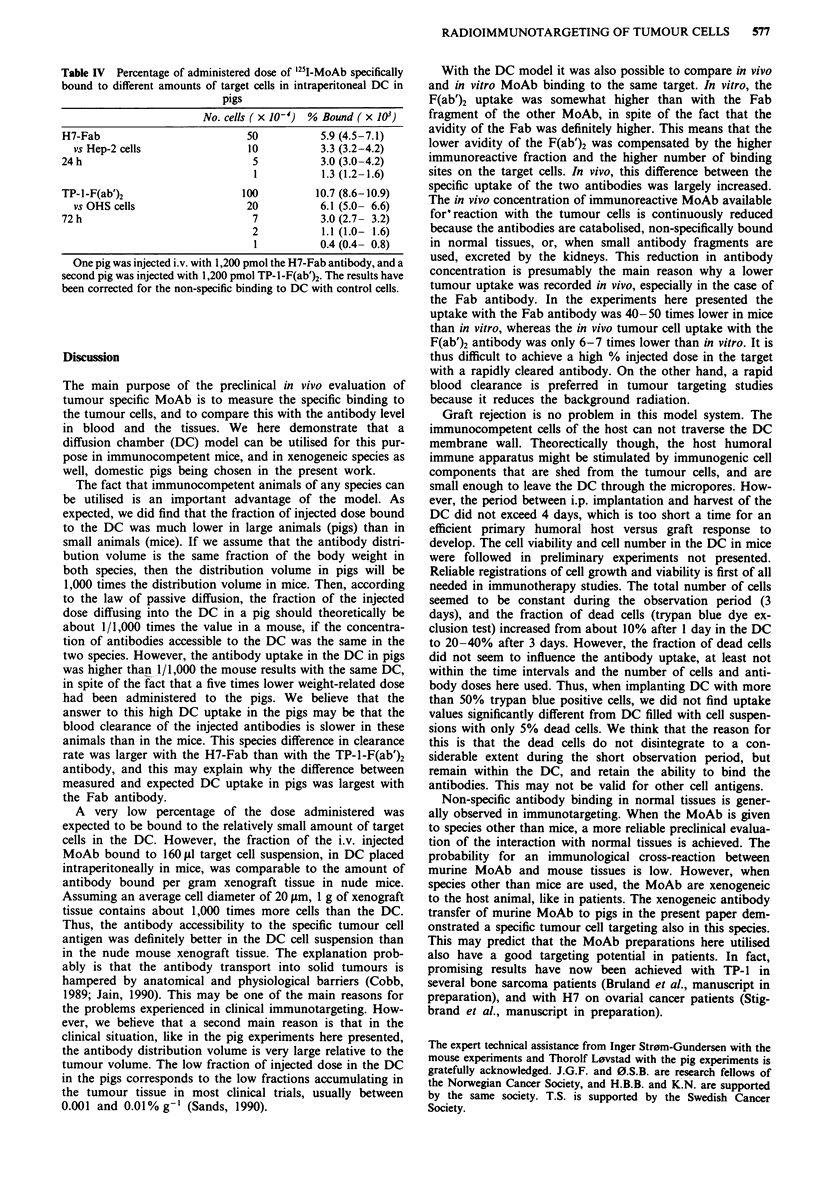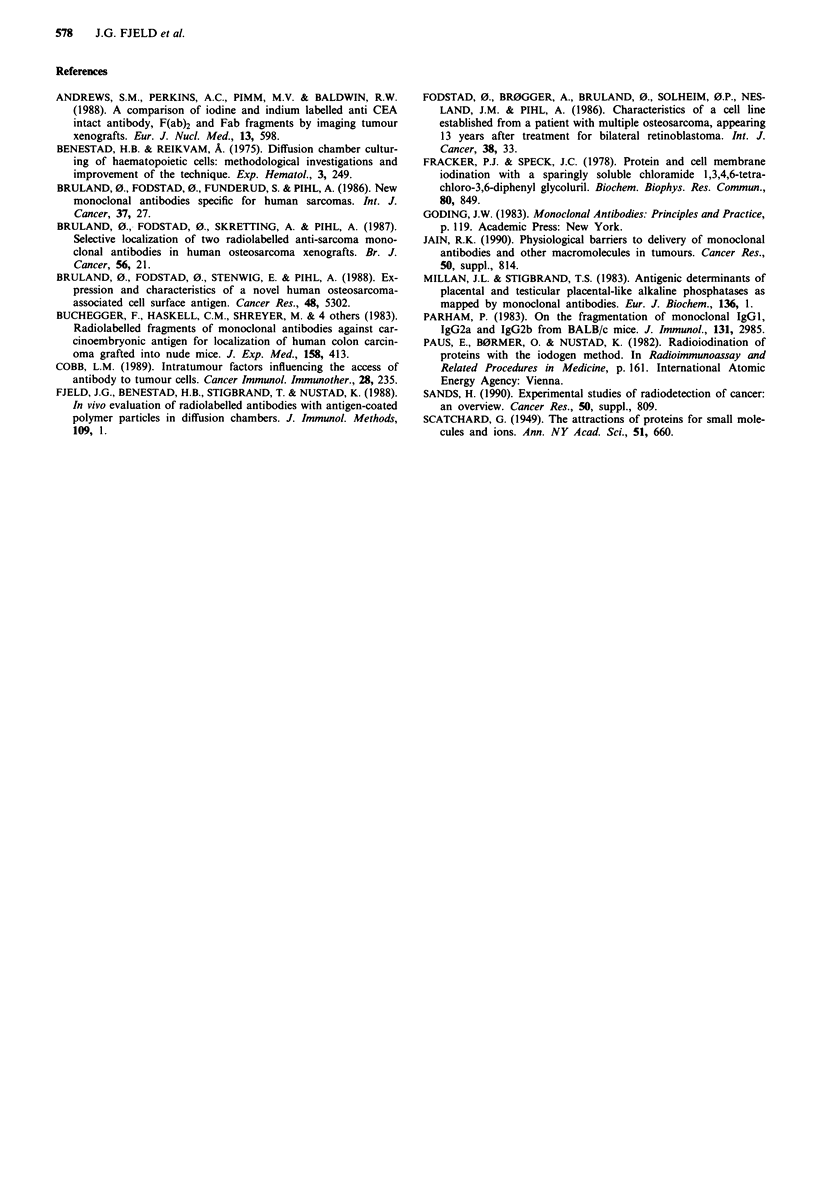# Radioimmunotargeting of human tumour cells in immunocompetent animals.

**DOI:** 10.1038/bjc.1990.332

**Published:** 1990-10

**Authors:** J. G. Fjeld, O. S. Bruland, H. B. Benestad, L. Schjerven, T. Stigbrand, K. Nustad

**Affiliations:** Central Laboratory, Norwegian Radium Hospital, Montebello, Oslo.

## Abstract

A tumour model system is reported that for many purposes may be an alternative to xenografted nude mice. The model allows immunotargeting of human tumour cells in immunocompetent animals. The target cells are contained in i.p. diffusion chambers (DC) with micropore membrane walls that are permeable to molecules, including the cell specific monoclonal antibodies (MoAb), but impermeable to cells. Thus, the tumour cells are protected from the host immunocompetent cells. In the work here presented the model was tested in immunocompetent mice and pigs, with tumour cells and antibody preparations that had demonstrated specific targeting in the nude mouse xenograft model. Hence, the DC were filled with cells from the human cell lines Hep-2 (expressing placental alkaline phosphatase, PLALP), or OHS (a sarcoma cell line), and the MoAb preparations injected i.v. were a 125I-labelled Fab fragment of the PLALP specific antibody H7, or a 125I-labelled F(ab')2 fragment of the sarcoma specific antibody TP-1. Specific targeting of the human tumour cells was demonstrated in both mice and pigs. The target: blood ratios were comparable in the two species, reaching a maximum of about 15 after 24 h with the Fab preparation, and a ratio of 25 after 72 h with the F(ab')2. The target uptake relative to injected dose was lower in pigs than in mice, but the difference between the two species was smaller than expected, presumably due to a slower antibody clearance in the pigs than in the mice. An artificial cell targeting system like this has several advantages in the search for solutions to many of the fundamental problems experienced in immunotargeting. Firstly, parallel binding experiments can be carried out in vitro with the same target. Because in vitro results are only influenced by the diffusion into the DC and the immunological binding characteristics of the antibodies, targeting differences between antibody preparations due to these factors can then be distinguished from differences due to pharmacokinetical properties. Secondly, the animals can be implanted with any type and number of target cells, or with antigen negative control cells. Thirdly, and perhaps most important, the system opens a possibility for evaluation of the murine MoAb in xenogenic species, and this may predict the clinical targeting potential better than experiments on mice.


Br. J. Cancer (1990), 62, 573-578 © Macmillan Press Ltd., 1990

### Radioimmunotargeting of human tumour cells in immunocompetent animals

J.G. Fjeld[1], Ø.S. Bruland[2], H.B. Benestad[3], L. Schjerven[4], T. Stigbrand[5] & K. Nustad[1]

[1]Central Laboratory and [2]Department of Medical Oncology and Radiotherapy, The Norwegian Radium Hospital, Montebello, N-0310 Oslo 3; [3]Institute of Physiology, University of Oslo, Karl Johansgt. 47, N-0162 Oslo 1; [4]Experimental Animal Department, The National Hospital of Norway, N-0027, Oslo 1, Norway; and [5]Department of Physiological Chemistry, University of Umeå, S-901 87 Umeå, Sweden.

**Summary** A tumour model system is reported that for many purposes may be an alternative to xenografted nude mice. The model allows immunotargeting of human tumour cells in immunocompetent animals. The target cells are contained in i.p. diffusion chambers (DC) with micropore membrane walls that are permeable to molecules, including the cell specific monoclonal antibodies (MoAb), but impermeable to cells. Thus, the tumour cells are protected from the host immunocompetent cells. In the work here presented the model was tested in immunocompetent mice and pigs, with tumour cells and antibody preparations that had demonstrated specific targeting in the nude mouse xenograft model. Hence, the DC were filled with cells from the human cell lines Hep-2 (expressing placental alkaline phosphatase, PLALP), or OHS (a sarcoma cell line), and the MoAb preparations injected i.v. were a $^{125}$I-labelled Fab fragment of the PLALP specific antibody H7, or a $^{125}$I-labelled F(ab')$_2$ fragment of the sarcoma specific antibody TP-1. Specific targeting of the human tumour cells was demonstrated in both mice and pigs. The target:blood ratios were comparable in the two species, reaching a maximum of about 15 after 24 h with the Fab preparation, and a ratio of 25 after 72 h with the F(ab')$_2$. The target uptake relative to injected dose was lower in pigs than in mice, but the difference between the two species was smaller than expected, presumably due to a slower antibody clearance in the pigs than in the mice. An artificial cell targeting system like this has several advantages in the search for solutions to many of the fundamental problems experienced in immunotargeting. Firstly, parallel binding experiments can be carried out *in vitro* with the same target. Because *in vitro* results are only influenced by the diffusion into the DC and the immunological binding characteristics of the antibodies, targeting differences between antibody preparations due to these factors can then be distinguished from differences due to pharmacokinetical properties. Secondly, the animals can be implanted with any type and number of target cells, or with antigen negative control cells. Thirdly, and perhaps most important, the system opens a possibility for evaluation of the murine MoAb in xenogeneic species, and this may predict the clinical targeting potential better than experiments on mice.

Radioimmunotargeting is based on the high specificity of antigen-antibody reactions. The method can be used for diagnostic as well as therapeutic purposes, depending on the dose and the physical properties of the radioactive label. Tumour visualisation is often hampered by insufficient tumour uptake and a too high antibody level in blood and non-tumour tissues. When used for immunotherapy, a such unfavourable antibody distribution would given an insufficient radioactivity dose in the tumour, and a detrimental dose to normal tissues.

Preclinical *in vivo* procedures with experimental animals, most often xenografted nude mice, are currently performed to restrict patient trials to the most promising anti-tumour antibodies. However, the nude mouse xenograft system has some important limitations. Firstly, the predictive value of the nude mouse model is reduced because most MoAb are produced in mice, so that the model does not test a xenogeneic antibody transfer, as occurs when murine MoAb are injected into patients. A large number of MoAb predict an excellent targeting potential when testing in the nude mouse model, whereas disappointingly few antibodies seem to give satisfactory tumour targeting in patients. Secondly, the growth rate of xenografted tumours varies between individual animals, giving heterogeneous tumour sizes that increase the variation in the experimental results. Thirdly, athymic nude mice are expensive, and require expensive housing conditions to be protected from infections.

Recently, a novel method for preclinical *in vivo* evaluation of anti-tumour MoAb was published from our institution. The method is based on diffusion chambers implanted in the peritoneal cavity of conventional laboratory animals, and was successfully used for targeting of antigen-coated polymer particles (Fjeld *et al.*, 1988). In the report here presented, human tumour cells rather than antigen-coated polymer particles were used. Importantly, with this DC tumour model it is possible to carry out targeting studies with the same MoAb preparations and tumour cell lines in any animal species large enough to carry the DC. Thus, to be able to compare xenogeneic and allogeneic transfer of the anti-tumour antibodies, we have chosen both domestic pigs and conventional laboratory mice as experimental animals. Antibody fragments do most often show improved tumour imaging characteristics compared with intact antibodies (Buchegger *et al.*, 1983; Andrew *et al.*, 1988). Two fragmented MoAb will be tested in the DC model in this paper.

### Materials and methods

### Animals

*Mice* The mice were randomly bred females (NMRI/Bom), 8-12 weeks of age and 20-25 g in weight.

*Pigs* Female domestic pigs were used. These animals were 4 months of age and 20 kg in weight.

### Diffusion chambers (DC)

*Preparation of DC* We used the same type of chambers as described in our previous work on DC with antigen-coated polymer particles (Fjeld *et al.*, 1988). The DC consisted of two micropore membranes (Millipore GSWP, mean pore diameter 0.22 μm) heat sealed to both sides of a 2 mm thick acrylic plastic ring, outer diameter 13 mm (Benestad & Reikvam, 1975). The DC were filled with 160 μl suspension of one of the two tumour cell lines (see below) in Dulbecco's Modified Eagle Medium (Gibco Limited, UK) with 10% FCS, or

Correspondence: J.G. Fjeld.
Received 21 March 1989; and in revised form 10 April 1990.

with the cell medium only as a cell free control. Each DC contained 160 μl cell suspension, and the number of cells in mice was $2 \times 10^5$ cells per DC, and from $10^4$ to $10^6$ cells per DC in pigs. When viability was tested with the trypan blue dye exclusion test, the implanted cell suspensions contained about 5% dead cells, increasing to about 10% after 24 h, and 20–40% after 72 h. There was no significant change in the total number of cells (dead + viable) during the observation period. Each DC was marked with waterproof ink, indicating the chamber content.

*DC implantation in mice* Two DC were i.p. implanted in each mouse through a midline ventral incision; one target DC and one control. The operation was carried out under ether anaesthesia the day before antibody injection. The wounds were closed with metal clips, and the mice were kept in conventional cages with free access to food and water.

*DC implantation in pigs* The animals were preoperatively sedated with azaperone, followed by anaesthesia with metomidate chloride i.v. during the operation. The pigs received 30 DC each, through a small midline laparothomy. These chambers contained different dilutions of cells, as described above, with triplicates for each dilution. The wounds were suturated, and the animals were kept in conventional cages with free access to food and water.

### *Tumour cell lines*

Two cell lines of human origin were used. The cervix cancer cell line HeLa, strain Hep-2, expresses placental alkaline phosphatase (PLALP), and these cells bind the monoclonal antibody (MoAb) H7. The second cell line was the osterosarcoma line OHS, which has been established at the Norwegian Radium Hospital (Fodstad *et al.*, 1986), and the sarcoma specific MoAb TP-1 binds to OHS epitopes.

### *Antibodies*

*The fragmented antibody preparations used* The monoclonal antibody H7 (IgG2a) recognises the three common allelic variants of PLALP (Millan & Stigbrand, 1983). The monoclonal antibody TP-1 (IgG2a) (Bruland *et al.*, 1986) is specific for an epitope on a sarcoma associated antigen (Bruland *et al.*, 1988). The immunoglobulin fractions were separated from ascites with affinity chromatography on protein-A Sepharose columns (Pharmacia, Sweden), and purity was controlled with Fast Protein Liquid Chromatography (FPLC, Pharmacia, Sweden), or polyacrylamide gel electrophoresis, as earlier described for both H7 (Fjeld *et al.*, 1988), and TP-1 (Bruland *et al.*, 1987).

The two MoAb were enzymatically digested before they were used *in vivo*. H7 was enzymatically fragmented to Fab with papain (Goding, 1983; Fjeld *et al.*, 1988), and TP-1 to $F(ab')_2$ with pepsin (Parham, 1983; Bruland *et al.*, 1987).

*Iodination of antibodies* The fragmented antibodies were $^{125}I$-labelled with Iodogen (Pierce, USA) as oxidant (Fracker & Speck, 1978). We followed a standard procedure established in our laboratory (Paus *et al.*, 1982).

*Quality control of the iodinated antibodies* Binding assays were carried out by mixing 50 μl of $^{125}I$-labelled MoAb dilution with 50 μl of serial dilutions of Hep-2 or OHS cells, respectively. The reactants had been diluted in PBS with 0.1% BSA. The reaction mixtures were incubated at 20°C for at least 3 h with continuous shaking. Free, labelled MoAb were then separated from cell-bond MoAb by washing twice with 1 ml of the dilution buffer. The cells were collected by centrifugation, and the bound radioactivity was counted in a gamma-counter. The immunoreactive fraction (F) of the labelled antibody preparation, the antibody association constant (Ka) and the number of binding sites (S) per cell were calculated from the experimental data on the assumption that the reactions obey the first order law of mass action. A computerised iteration technique was utilised to achieve the parameter values that give optimal fitness of the experimental values with the mass action law (Fjeld & Skretting, manuscript in preparation). Shortly, the assumptions made by us for the reaction between antigen and antibody were the same as the assumptions for most of the plot methods utilised, for example the Scatchard plot (Scatchard, 1949), i.e. a reversible bimolecular reaction following second order kinetics. The difference is that a computational fitting procedure was utilised in exchange for a graphical curve fitting by the eye. The procedure was limited to experimental values giving between 30 and 100% binding of the immunoreactive fraction of the $^{125}I$-MoAbs.

*Intravenous injection in mice* The next day after the DC implantation, the mice were injected i.v. (tail vein) with 6 pmol $^{125}I$-labelled antibodies per mouse (equivalent to 0.3 μg H7-Fab; or 0.6 μg TP-1-$F(ab')_2$) diluted in 0.9% NaCl with 0.1% normal mouse serum (200 μl per mouse).

*Intravenous injection in pigs* $^{125}I$-labelled MoAb was injected i.v. (ear vein) immediately after the DC implantation, while the pigs were still in deep anaesthesia. Each pig received 1,200 pmol antibody (equivalent to 55 μg H7-Fab; or 120 μg TP-1-$F(ab')_2$) in a volume of 5 ml per animal.

### *DC and tissue harvest*

*Mice* Blood, tissue specimens, and the DC were collected from animals killed by ether overdosage at various time intervals during the next 3 days after the injection. The radioactivity in the whole DC with its tumour cell content, and in blood samples and tissue specimens were counted in a gamma counter.

*Pigs* The pigs remained anaesthetised during the first 2–3 h period after the DC implantation, because multiple blood samples were collected during this period. The blood was collected from the ear vein, or from veins in the thoracic outlet. They were killed by an overdose of phenobarbitone after a period of 1–3 days, depending on which antibody preparation that had been injected. Like in the mouse experiments, the distribution and tumour cell targeting of the labelled antibodies were studied by recording in a gamma-counter the radioactivity in blood, various tissues, and all the different DC in the peritoneal cavity.

### Results

### *Binding characteristics of the antibodies and the tumour cells*

The two $^{125}I$-labelled antibody preparations chosen for the targeting experiments were first tested *in vitro* against cell suspensions from the cell lines Hep-2 and OHS. The Fab fragment of the MoAb H7 (H7-Fab) did bind to Hep-2, and the $F(ab')_2$ fragment of TP-1 (TP-1-$F(ab')_2$) to OHS (Figure 1). The non-specific binding of H7-Fab to OHS and of TP-1-$F(ab')_2$ to Hep-2 was only about 1% of total radioactivity (not shown in Figure 1).

The binding parameters of the iodinated preparations were then estimated, using a mathematical model derived from the first order law of mass action. A computerised non-linear least-squares fit to the experimental data yielded the binding parameters (Fjeld & Skretting, manuscript in preparation). The estimated association constant of H7-Fab was closed to ten times the association constant of the TP-1-$F(ab')_2$, whereas the immunoreactive fraction as well as the number of binding sites per target cell was higher for the TP-1-$F(ab')_2$ than for H7-Fab (Table I).

### *Immunotargeting of tumour cells in immunocompetent mice*

The two antibody preparations had previously been used in the nude mouse xenograft model system (Bruland *et al.*,

**Figure 1** *In vitro* binding of $^{125}$I-labelled H7-Fab to Hep-2 cell suspensions (O- - -O), and of $^{125}$I-labelled TP-1-F(ab')$_2$ to OHS (●——●). A constant amount of $^{125}$I-labelled antibody in 50 µl was added to 50 µl aliquots of serial dilutions of target cells. After incubation at 20°C for at least 3 h, followed by two washes, the amount of cell bound radioactivity was counted. The results have been corrected for non-specific binding to the other cell line, and the antibodies were highly selective for their target cells, with only about 1% non-specific binding. Medians for triplicates.

**Table I** Binding parameters for the $^{125}$I-MoAb preparations

| | *Immunoreactive fraction (%)* | *Association constant ($l\,mol^{-1}$)* | *No. sites per cell ($\times 10^{-5}$)* |
|---|---|---|---|
| TP-1-F(ab')$_2$ *vs* OHS cells | 83 | $7.4 \times 10^8$ | 6.4 |
| H7-Fab *vs* Hep-2 cells | 57 | $6.0 \times 10^9$ | 4.0 |

The binding assay and the calculation of the parameters was carried out as described in Materials and methods. The experimental results used in the calculations are presented in Figure 1.

1987; Fjeld, unpublished results). Moreover, the H7 preparation had also shown specific targeting of antigen coated polymer particles in normal mice (Fjeld *et al.*, 1988).

The *in vivo* uptake of a radiolabelled antibody in its specific target depends, among several other factors, on the antibody concentration in the blood. Thus, the DC targeting experiments were preceded by blood clearance studies with the anti-tumour antibodies in immunocompetent mice (Figure 2). The Fab fragment of H7 was, as expected, more rapidly cleared from the blood circulation than the larger F(ab')$_2$ fragment of TP-1. The next day after the injection the blood level of both antibodies was low, but significantly different, with a value of 0.1% injected dose for H7-Fab and 5% for TP-1-F(ab')$_2$. It was necessary to wait for 2 more days before the level of the TP-1 preparation had decreased to the low level reached by the H7 preparation after only 1 day.

Based on these blood clearance studies, it was decided to compare the biodistribution and tumour cell targeting 24 and 72 h after the antibody injection. Each mouse carried two DC, one filled with $2 \times 10^5$ OHS cells, and the second with the same amount of Hep-2 cells. The results demonstrated that the anti-sarcoma antibody TP-1-F(ab')$_2$ was specifically bound to DC filled with cells from the sarcoma cell line OHS, whereas the anti-PLALP antibody H7-Fab was specifically bound to DC containing the PLALP expressing Hep-2 cells (Figure 3). Moreover, the DC:blood ratio with the TP-1 preparation was significantly increased in the period from 1 to 3 days. In contrast, the DC:blood ratio with the H7 preparation decreased from day 1 to day 3. The explanation to the latter seemed to be that there had been a net release of radioactivity from the target in the period between day 1 and day 3 (Table II), whereas the blood level remained almost unchanged (Figure 2). The explanation to the increased target:blood ratio with TP-1-F(ab')$_2$ seemed to be no change, or a minor increase, in the net radioactivity uptake in the DC between 1 and 3 days (Table II), whereas the blood level had decreased during the same period (Figure 2).

**Figure 2** Blood concentrations of $^{125}$I-labelled H7-Fab (O- - -O) and TP-1-F(ab')$_2$ (●——●) injected i.v. on mice. Single observations.

**Figure 3** Biodistribution and human tumour cell immunotargeting in mice 24 h and 72 h after antibody injection i.v. Each mouse carried two DC, one filled with human tumour cell suspension, and the other with control cells. The DC contained $2 \times 10^5$ cells in a volume of 160 µl cell medium, and the radioactivity in tissue specimens and the DC were therefore related to the radioactivity in 160 µl blood. Medians and ranges for groups of three DC are shown. There were only minor differences between the tissue results, and these ranges were therefore not drawn.

**Table II** Percentage of administered dose of $^{125}$I-MoAb specifically bound to human target cells in intraperitoneal DC in mice

| *Time after i.v. injection (h)* | *TP-1-F(ab')$_2$* | *H7-Fab* | *Ratio TP-1-F(ab')$_2$:H7-Fab* |
|---|---|---|---|
| 24 | 1.8% (1.4–1.9) | 0.21% (0.20–0.35) | 8.6 |
| 72 | 1.8% (1.6–2.4) | 0.13% (0.09–0.16) | 13.8 |

Each DC contained $2 \times 10^5$ target or control cells. Each mouse, with one target and one control DC, was injected i.v. with 6 pmol antibody. The results were corrected for the non-specific binding to control DC. Medians and ranges for groups of three DC.

The uptake in DC filled with the cell medium only was not significantly different from the DC with the control cells (results not shown). Thus, the radioactivity in the control DC did mostly represent non-specific binding to the chamber components, and unbound antibodies in the fluid within the DC.

### In vitro *DC studies parallel to the mouse experiments*

With the artificial tumour system here presented, a comparison between *in vitro* and *in vivo* uptake could be performed.

**Table III** *In vitro* targeting of DC with human tumour cells

| *Incubation time (h)* | *TP-1-F(ab')₂* | *H7-Fab* | *Ratio TP-1-F(ab')₂:H7-Fab* |
|---|---|---|---|
| 24 | 10.9% (9.2–11.4) | 7.8% (7.2–7.9) | 1.4 |
| 72 | 12.9% (10.8–13.7) | 6.9% (3.9–7.8) | 1.9 |

Test tubes with 10 ml cell medium and 0.3 pmol $^{125}$I-labelled antibody added were used. Each tube contained three DC with $2 \times 10^5$ target cells, and three DC with the same number of control cells. The tubes were rotated end over end at 37°C. Percentage of added dose bound to the target DC is shown, corrected for the non-specific binding to control DC. Medians and ranges for three DC.

It is then possible during evaluation of the immunotargeting capacity of an antibody, to distinguish between pharmacokinetical and immunological factors. *In vitro*, the specific DC binding was somewhat higher with TP-1 than with H7; after 24 h there was a 1.4 times higher TP-1 uptake, increasing to 1.9 times at 72 h (Table III). This minor difference between the two antibodies *in vitro* was in contrast to the results in the peritoneal cavity of mice, where a definitely larger difference was recorded; after 24 h there was a 8.6 times higher *in vivo* uptake of the TP-1 antibody than of H7, increasing to a ratio of 13.8 after 72 h (Table II). Thus, when comparing the targeting potential of these two antibodies, it seems that most of the difference between their tumour cell uptake was caused by pharmacokinetical differences between Fab and F(ab')$_2$, and only a minor part was due to different immunological binding characteristics.

### *Immunotargeting of tumour cells in domestic pigs*

Like in the mouse experiments, the blood clearance of the two antibody preparations was recorded in the pigs before the targeting experiments were carried out (Figure 4). Guided by these clearance curves, a 24 h period between MoAb injection and DC harvest was chosen for Fab, and a 72 h period for F(ab')$_2$, e.g. the same time intervals that had given optimal targeting in the mice. The clearance rate of both antibodies was slower in the pigs than in mice. A higher blood level of antibodies in the pigs should enhance the tumour uptake in this species. With the intention to decrease the effect of this difference in clearance rate on the concentration of antibody available for the tumour cells during the study, the antibody doses given to the pigs were only increased 200 times relative to the doses injected on the mice, in spite of the fact that the body weight ratio between the two species was about 1,000.

The targeting experiments showed that the binding of both antibodies to the specific target did increase with increasing number of tumour cells (Figures 5 and 6, Table IV). Analogous to an *in vitro* assay, the degree of increase in the antibody uptake with increasing amount of tumour cells reflects partly the antibody avidity for the cells, and partly the number of specific binding sites on the cell surfaces relative to the amount of antibody available.

When comparing the results in mice and pigs, we found that the DC:blood ratios recorded in the pigs (Figures 5 and 6) were of the same order of magnitude as the ratios previously achieved in mice (Figure 3), whereas the DC uptake relative to injected dose was definitely lower in the pigs (Table IV) than in the mice (Table II). The fraction of injected TP-1-F(ab')$_2$ bound in the pig DC with $2 \times 10^5$ cells was 1/300 times the mouse DC uptake ($2 \times 10^5$ cells). With H7-Fab the DC with half the cell number (i.e. $10^5$ cells) did bind as much as 1/60 the uptake in the mice. Thus, the DC uptake values in pigs were definitely higher than expected from the mouse results.

Several pig tissue specimens were explored for their radioactivity uptake, without being presented in Figure 5 and Figure 6. There was an overall low radioactivity level in all these tissues. Different bone and cartilage specimens were carefully controlled for uptake of the anti-sarcoma antibody, and the level was low also in these tissues.

**Figure 4** Blood concentration of $^{125}$I-labelled H7-Fab (O- - -O) and TP-1-F(ab')$_2$ (●——●) injected i.v. on pigs. Single observations.

**Figure 5** Biodistribution and immunotargeting of human tumour cells in a pig 24 h after i.v. injection of $^{125}$I-labelled H7-Fab. Each bar represents the median and range for 3 DC, or single values for tissue specimens.

**Figure 6** Biodistribution and human tumour cell targeting of $^{125}$I-labelled TP-1-F(ab')$_2$ in a pig 72 h after i.v. injection. See legend to Figure 5.

**Table IV** Percentage of administered dose of [125]I-MoAb specifically bound to different amounts of target cells in intraperitoneal DC in pigs

| | *No. cells* ( $\times 10^{-4}$ ) | *% Bound* ( $\times 10^{3}$ ) |
|---|---|---|
| H7-Fab | 50 | 5.9 (4.5–7.1) |
| *vs* Hep-2 cells | 10 | 3.3 (3.2–4.2) |
| 24 h | 5 | 3.0 (3.0–4.2) |
| | 1 | 1.3 (1.2–1.6) |
| TP-1-F(ab')$_2$ | 100 | 10.7 (8.6–10.9) |
| *vs* OHS cells | 20 | 6.1 (5.0– 6.6) |
| 72 h | 7 | 3.0 (2.7– 3.2) |
| | 2 | 1.1 (1.0– 1.6) |
| | 1 | 0.4 (0.4– 0.8) |

One pig was injected i.v. with 1,200 pmol the H7-Fab antibody, and a second pig was injected with 1,200 pmol TP-1-F(ab')$_2$. The results have been corrected for the non-specific binding to DC with control cells.

### Discussion

The main purpose of the preclinical *in vivo* evaluation of tumour specific MoAb is to measure the specific binding to the tumour cells, and to compare this with the antibody level in blood and the tissues. We here demonstrate that a diffusion chamber (DC) model can be utilised for this purpose in immunocompetent mice, and in xenogeneic species as well, domestic pigs being chosen in the present work.

The fact that immunocompetent animals of any species can be utilised is an important advantage of the model. As expected, we did find that the fraction of injected dose bound to the DC was much lower in large animals (pigs) than in small animals (mice). If we assume that the antibody distribution volume is the same fraction of the body weight in both species, then the distribution volume in pigs will be 1,000 times the distribution volume in mice. Then, according to the law of passive diffusion, the fraction of the injected dose diffusing into the DC in a pig should theoretically be about 1/1,000 times the value in a mouse, if the concentration of antibodies accessible to the DC was the same in the two species. However, the antibody uptake in the DC in pigs was higher than 1/1,000 the mouse results with the same DC, in spite of the fact that a five times lower weight-related dose had been administered to the pigs. We believe that the answer to this high DC uptake in the pigs may be that the blood clearance of the injected antibodies is slower in these animals than in the mice. This species difference in clearance rate was larger with the H7-Fab than with the TP-1-F(ab')$_2$ antibody, and this may explain why the difference between measured and expected DC uptake in pigs was largest with the Fab antibody.

A very low percentage of the dose administered was expected to be bound to the relatively small amount of target cells in the DC. However, the fraction of the i.v. injected MoAb bound to 160 µl target cell suspension, in DC placed intraperitoneally in mice, was comparable to the amount of antibody bound per gram xenograft tissue in nude mice. Assuming an average cell diameter of 20 µm, 1 g of xenograft tissue contains about 1,000 times more cells than the DC. Thus, the antibody accessibility to the specific tumour cell antigen was definitely better in the DC cell suspension than in the nude mouse xenograft tissue. The explanation probably is that the antibody transport into solid tumours is hampered by anatomical and physiological barriers (Cobb, 1989; Jain, 1990). This may be one of the main reasons for the problems experienced in clinical immunotargeting. However, we believe that a second main reason is that in the clinical situation, like in the pig experiments here presented, the antibody distribution volume is very large relative to the tumour volume. The low fraction of injected dose in the DC in the pigs corresponds to the low fractions accumulating in the tumour tissue in most clinical trials, usually between 0.001 and 0.01% $g^{-1}$ (Sands, 1990).

With the DC model it was also possible to compare *in vivo* and *in vitro* MoAb binding to the same target. *In vitro*, the F(ab')$_2$ uptake was somewhat higher than with the Fab fragment of the other MoAb, in spite of the fact that the avidity of the Fab was definitely higher. This means that the lower avidity of the F(ab')$_2$ was compensated by the higher immunoreactive fraction and the higher number of binding sites on the target cells. *In vivo*, this difference between the specific uptake of the two antibodies was largely increased. The *in vivo* concentration of immunoreactive MoAb available for reaction with the tumour cells is continuously reduced because the antibodies are catabolised, non-specifically bound in normal tissues, or, when small antibody fragments are used, excreted by the kidneys. This reduction in antibody concentration is presumably the main reason why a lower tumour uptake was recorded *in vivo*, especially in the case of the Fab antibody. In the experiments here presented the uptake with the Fab antibody was 40–50 times lower in mice than *in vitro*, whereas the *in vivo* tumour cell uptake with the F(ab')$_2$ antibody was only 6–7 times lower than *in vitro*. It is thus difficult to achieve a high % injected dose in the target with a rapidly cleared antibody. On the other hand, a rapid blood clearance is preferred in tumour targeting studies because it reduces the background radiation.

Graft rejection is no problem in this model system. The immunocompetent cells of the host can not traverse the DC membrane wall. Theorectically though, the host humoral immune apparatus might be stimulated by immunogenic cell components that are shed from the tumour cells, and are small enough to leave the DC through the micropores. However, the period between i.p. implantation and harvest of the DC did not exceed 4 days, which is too short a time for an efficient primary humoral host versus graft response to develop. The cell viability and cell number in the DC in mice were followed in preliminary experiments not presented. Reliable registrations of cell growth and viability is first of all needed in immunotherapy studies. The total number of cells seemed to be constant during the observation period (3 days), and the fraction of dead cells (trypan blue dye exclusion test) increased from about 10% after 1 day in the DC to 20–40% after 3 days. However, the fraction of dead cells did not seem to influence the antibody uptake, at least not within the time intervals and the number of cells and antibody doses here used. Thus, when implanting DC with more than 50% trypan blue positive cells, we did not find uptake values significantly different from DC filled with cell suspensions with only 5% dead cells. We think that the reason for this is that the dead cells do not disintegrate to a considerable extent during the short observation period, but remain within the DC, and retain the ability to bind the antibodies. This may not be valid for other cell antigens.

Non-specific antibody binding in normal tissues is generally observed in immunotargeting. When the MoAb is given to species other than mice, a more reliable preclinical evaluation of the interaction with normal tissues is achieved. The probability for an immunological cross-reaction between murine MoAb and mouse tissues is low. However, when species other than mice are used, the MoAb are xenogeneic to the host animal, like in patients. The xenogeneic antibody transfer of murine MoAb to pigs in the present paper demonstrated a specific tumour cell targeting also in this species. This may predict that the MoAb preparations here utilised also have a good targeting potential in patients. In fact, promising results have now been achieved with TP-1 in several bone sarcoma patients (Bruland *et al.*, manuscript in preparation), and with H7 on ovarial cancer patients (Stigbrand *et al.*, manuscript in preparation).

The expert technical assistance from Inger Strøm-Gundersen with the mouse experiments and Thorolf Løvstad with the pig experiments is gratefully acknowledged. J.G.F. and Ø.S.B. are research fellows of the Norwegian Cancer Society, and H.B.B. and K.N. are supported by the same society. T.S. is supported by the Swedish Cancer Society.

### References

ANDREWS, S.M., PERKINS, A.C., PIMM, M.V. & BALDWIN, R.W. (1988). A comparison of iodine and indium labelled anti CEA intact antibody, $F(ab)_2$ and Fab fragments by imaging tumour xenografts. *Eur. J. Nucl. Med.*, **13**, 598.

BENESTAD, H.B. & REIKVAM, Å. (1975). Diffusion chamber culturing of haematopoietic cells: methodological investigations and improvement of the technique. *Exp. Hematol.*, **3**, 249.

BRULAND, Ø., FODSTAD, Ø., FUNDERUD, S. & PIHL, A. (1986). New monoclonal antibodies specific for human sarcomas. *Int. J. Cancer*, **37**, 27.

BRULAND, Ø., FODSTAD, Ø., SKRETTING, A. & PIHL, A. (1987). Selective localization of two radiolabelled anti-sarcoma monoclonal antibodies in human osteosarcoma xenografts. *Br. J. Cancer*, **56**, 21.

BRULAND, Ø., FODSTAD, Ø., STENWIG, E. & PIHL, A. (1988). Expression and characteristics of a novel human osteosarcoma-associated cell surface antigen. *Cancer Res.*, **48**, 5302.

BUCHEGGER, F., HASKELL, C.M., SHREYER, M. & 4 others (1983). Radiolabelled fragments of monoclonal antibodies against carcinoembryonic antigen for localization of human colon carcinoma grafted into nude mice. *J. Exp. Med.*, **158**, 413.

COBB, L.M. (1989). Intratumour factors influencing the access of antibody to tumour cells. *Cancer Immunol. Immunother.*, **28**, 235.

FJELD, J.G., BENESTAD, H.B., STIGBRAND, T. & NUSTAD, K. (1988). *In vivo* evaluation of radiolabelled antibodies with antigen-coated polymer particles in diffusion chambers. *J. Immunol. Methods*, **109**, 1.

FODSTAD, Ø., BRØGGER, A., BRULAND, Ø., SOLHEIM, Ø.P., NESLAND, J.M. & PIHL, A. (1986). Characteristics of a cell line established from a patient with multiple osteosarcoma, appearing 13 years after treatment for bilateral retinoblastoma. *Int. J. Cancer*, **38**, 33.

FRACKER, P.J. & SPECK, J.C. (1978). Protein and cell membrane iodination with a sparingly soluble chloramide 1,3,4,6-tetrachloro-3,6-diphenyl glycoluril. *Biochem. Biophys. Res. Commun.*, **80**, 849.

GODING, J.W. (1983). *Monoclonal Antibodies: Principles and Practice*, p. 119. Academic Press: New York.

JAIN, R.K. (1990). Physiological barriers to delivery of monoclonal antibodies and other macromolecules in tumours. *Cancer Res.*, **50**, suppl., 814.

MILLAN, J.L. & STIGBRAND, T.S. (1983). Antigenic determinants of placental and testicular placental-like alkaline phosphatases as mapped by monoclonal antibodies. *Eur. J. Biochem.*, **136**, 1.

PARHAM, P. (1983). On the fragmentation of monoclonal IgG1, IgG2a and IgG2b from BALB/c mice. *J. Immunol.*, **131**, 2985.

PAUS, E., BØRMER, O. & NUSTAD, K. (1982). Radioiodination of proteins with the iodogen method. In *Radioimmunoassay and Related Procedures in Medicine*, p. 161. International Atomic Energy Agency: Vienna.

SANDS, H. (1990). Experimental studies of radiodetection of cancer: an overview. *Cancer Res.*, **50**, suppl., 809.

SCATCHARD, G. (1949). The attractions of proteins for small molecules and ions. *Ann. NY Acad. Sci.*, **51**, 660.